# Synthetic Peucedanocoumarin IV Prevents α-Synuclein Neurotoxicity in an Animal Model of Parkinson’s Disease

**DOI:** 10.3390/ijms23158618

**Published:** 2022-08-03

**Authors:** Heejeong Kim, Han-Joo Maeng, Ji Hun Kim, Jin-Ha Yoon, Yohan Oh, Seung-Mann Paek, Yunjong Lee

**Affiliations:** 1 Department of Pharmacology, Samsung Biomedical Research Institute, Sungkyunkwan University School of Medicine, Suwon 16419, Korea; kimhj9301@skku.edu (H.K.); slswhdwkd@skku.edu (J.H.K.); 2 College of Pharmacy, Gachon University, Incheon 21936, Korea; hjmaeng@gachon.ac.kr (H.-J.M.); jinha89@daum.net (J.-H.Y.); 3 Department of Biomedical Science, Graduate School of Biomedical Science and Engineering, Hanyang University, Seoul 04763, Korea; yoh@hanyang.ac.kr; 4 Department of Biochemistry and Molecular Biology, College of Medicine, Hanyang University, Seoul 04763, Korea; 5 College of Pharmacy and Research Institute of Pharmaceutical Sciences, Gyeongsang National University, Jinju Daero 501, Jinju 52828, Korea

**Keywords:** peucedanocoumarin IV, organic synthesis, Parkinson’s disease, pharmacokinetics, α-synuclein preformed fibril, dopaminergic cell loss

## Abstract

Pathological protein inclusion formation and propagation are the main causes of neuronal dysfunction in diverse neurodegenerative diseases; therefore, current disease-modifying therapeutic strategies have targeted this disease protein aggregation process. Recently, we reported that peucedanocoumarin III (PCiii) is a promising therapeutic compound with the ability to disaggregate α-synuclein inclusion and protect dopaminergic neurons in Parkinson’s disease (PD). Here, we found that *trans*-4′-acetyl-3′-tigloylkhellactone (racemic peucedanocoumarin IV [PCiv]), a structural isomer of PCiii with a higher synthetic yield presented a strong anti-aggregate activity to a degree comparable to that of PCiii. PCiv retained effective inhibitory function against β-sheet aggregate-mimic β23 cytotoxicities and potently prevented α-synucleinopathy in α-synuclein preformed fibril (PFF)-treated mice cortical neurons. In detailed pharmacokinetic profiling of PCiv, oral administration of PCiv in rats exhibited an approximately 97-min half-life and 10% bioavailability. Moreover, tissue distribution analysis revealed favorable profiles of brain penetration with a 6.4 brain-to-plasma concentration ratio. The therapeutic efficacy of PCiv was further evaluated in a sporadic PD mouse model with a combinatorial co-injection of α-synuclein preformed fibril and recombinant adeno-associated virus expressing α-synuclein. Motor dysfunctions induced in this combinatorial α-synucleinopathy PD mouse model was almost completely rescued by PCiv diet administration, and this therapeutic effect is consistent with the marked prevention of dopaminergic neuron loss and suppression of α-synuclein aggregation. Taken together, our translational study suggests that PCiv is advantageous as a therapeutic agent for neurodegenerative diseases, especially with its good synthetic yield, high brain distribution, and anti-aggregate activity. PCiv may be useful in the management of α-synuclein inclusion formation and propagation at different stages of PD.

## 1. Introduction

Parkinson’s disease (PD) is defined by α-synuclein aggregation and toxicity in ventral midbrain dopaminergic neurons, which is clinically diagnosed with characteristic motor symptoms, including bradykinesia, resting tremor, muscle rigidity, and postural instability [1,2]. Phosphorylated and misfolded α-synuclein is a major constituent of Lewy body inclusions and Lewy neurites in PD and related brain diseases such as α-synucleinopathy [3]. Approximately 80% of PD patients progressively manifest cognitive deficits over 15 years since the diagnosis of motor dysfunction, which is associated with the pathological propagation of Lewy pathologies from the midbrain towards the limbic and cortical brain areas [4]. Despite the extensive pathological involvement of diverse brain regions in PD patients, currently available therapies focus on mitigating overt symptoms mainly by supplementing the responsible neurotransmitters or augmenting specific receptor-regulated signaling pathways. However, these anti-symptomatic therapies fail to fundamentally target disease progression, which is thought to be mediated by α-synuclein pathology formation and propagation. For better and effective treatment of motor and diverse non-motor clinical symptoms in patients with PD, it is crucial to suppress α-synuclein aggregation or modulate signaling pathways that contribute to α-synuclein aggregation or propagation in PD environments.

Current approaches to targeting pathological α-synuclein include anti-α-synuclein antibody therapies and small compounds with the ability to inhibit α-synuclein aggregation. Current clinical trials of anti-α-synuclein therapies may hold promise in specifically blocking the propagation of α-synuclein aggregation or receptor-mediated activation of microglia directly by extracellular α-synuclein aggregates. However, the low brain permeability of antibodies and limited therapeutic effect on intracellular α-synuclein aggregation reflects the need to develop small compounds with favorable brain permeability profiles that inhibit intracellular α-synuclein aggregation. In this regard, our group reported that peucedanocoumarin III (PCiii) exhibits strong inhibitory activity against repeated β sheet aggregate mimic β23 induced toxicity [5,6]. β23 was previously invented to represent the general β sheet aggregate structure of many brain disease proteins, including amyloid beta, α-synuclein, and tau [7]. Both natural and synthetic PCiii compounds disaggregated and facilitated the proteasomal clearance of β23. Similarly, α-synuclein aggregation and neurotoxicity have been effectively prevented in a 6-OHDA-induced PD mouse model [6]. Although the PCiii organic synthesis protocol has been well established, and this compound possesses promising anti-aggregate therapeutic activity in neurodegenerative diseases, further studies on PCiii derivatives with equivalent or advanced pharmacological properties have not yet been conducted.

In this study, we found that the structural isomer of PCiii (named “PCiv”) retained anti-aggregate functions against general β sheet aggregate β23 and α-synuclein aggregates. PCiv had a higher synthetic yield and better brain penetration compared with PCiii. Importantly, PCiv was shown to be effective in treating motor deficits and dopaminergic α-synucleinopathy pathologies in a sporadic PD mouse model of combinatorial recombinant adeno-associated virus expressing α-synuclein (*rAAV-αSyn*) and α-synuclein preformed fibril (PFF) injection, indicating its translational value.

## 2. Results

### 2.1. Organic Synthesis of Peucedanocoumarin IV and Its Functional Evaluation

**Figure 1:**
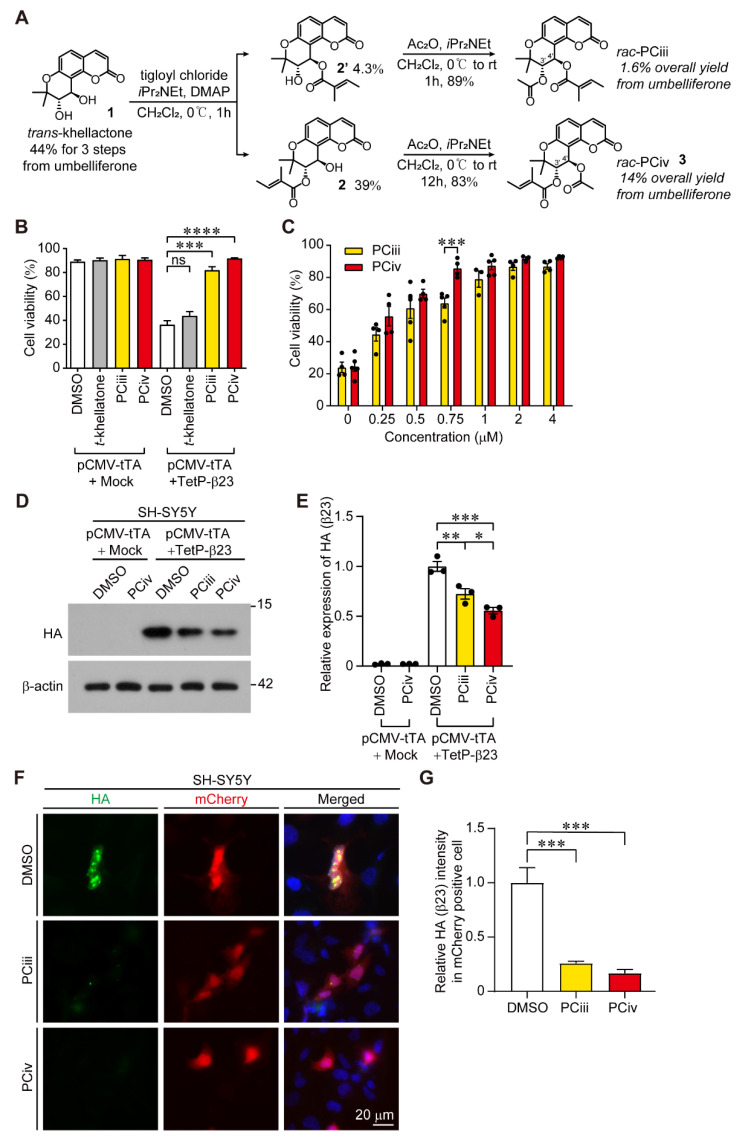
Peucedanocoumarin IV (PCiv) inhibits β23 aggregations and cytotoxicity in SH-SY5Y cells. (**A**) Scheme showing the detailed experimental steps in the organic synthesis of racemic PCiv and racemic PCiii (*rac*-PCiii, and *rac*-PCiv). Both PCiii and PCiv used in this study are racemic forms. (**B**) Trypan blue exclusion cell viability assessment in SH-SY5Y cells with Tet-Off-induced β23 expressions. β23 was expressed for 24 h using the Tet-off system. Further expression of β23 was halted by doxycycline (Dox) treatment with simultaneous treatment of the PCiii, PCiv, or their synthetic intermediate, *trans*-khellatone (*t*-khellatone, 1 μM 24 h). Cell viability was assessed 24 h after doxycycline treatment (*n* = 5 per group). (**C**) Synthetic PCiv’s protective effect on Tet-Off-expressed β23 toxicity in SH-SY5Y cells compared with peucedanocoumarin III (PCiii) determined by trypan blue exclusion assay. (**D**) The degradation of β23 monitored by Western blot at 37 h after doxycycline (200 ng/mL) treatment. The representative Western blot image of HA (β23) in SH-SY5Y cells treated with synthetic PCiii, synthetic PCiv (1 μM, 37 h), or DMSO as a vehicle. (**E**) Quantification of HA (β23) expression in SH-SY5Y cells from the indicated experimental groups in panel D (*n* = 3 experiments per group). (**F**) Representative immunofluorescence of steady state HA (β23) expression in SH-SY5Y cells expressing Tet-Off regulatable reporter mCherry and HA-tagged β23. HA-β23 was expressed for 24 h using the Tet-off system. Further expression of β23 was halted by Dox treatment in the presence of PCiii or PCiv (1 μM, 37 h). (**G**) Quantification of HA (β23) immunofluorescence signal in mCherry positive SH-SY5Y cells in each experimental group (*n* = 32 cells from three separate experiments per group). The data are expressed as means ± SEMs. * *p* < 0.05, ** *p* < 0.01, *** *p* < 0.001, and **** *p* < 0.0001, ANOVA test followed by Tukey’s post hoc analysis. Two-way ANOVA was used in panel C.

Previously we reported the synthesis of PCiii from commercially available umbelliferone via a straightforward, five-step route. Although this allowed for the preparation of PCiii for cell-based assays, its low chemical yield (1.6% after five steps) hampered further development [6]. In pursuit of an easier preparation of PCiii derivatives and their potent cellular activity, we turned our attention to synthetic intermediate **2**, which is a byproduct of PCiii synthesis (Figure 1A). With the employment of secondary alcohol **2** as a synthetic resource for further development, racemic *trans*-4′-acetyl-3′-tigloylkhellactone (*rac*-PCiv, **3**) was prepared after a simple acetylation process (Figure 1A and Supplementary Figure S1A,B). Compared to the synthetic efficiency of PCiii (1.6% after five steps), this method permitted easy access (14% after five steps) to large amounts of material on the gram scale (Figure 1A). A biological evaluation was performed using this material.

Since PCiii was previously screened out as an anti-aggregate with cytoprotective function against amyloid mimic β23, we sought to compare the cytoprotective function of PCiii and its structural isomer, PCiv, in SH-SY5Y cells with Tet-Off conditional expression of β23 using the trypan blue exclusion cell viability assay. Tet-Off pulsed expression of β23 for 24 h caused robust cytotoxicity, reducing cell viability below 50% in SH-SY5Y cells (Figure 1B). Consistent with a previous report [5,6], PCiii potently prevented β23-induced cytotoxicity. Interestingly, the structural isomer PCiv with greater synthetic yield also possessed nearly equivalent cytoprotective biological function against β23 cytotoxicities compared to PCiii (Figure 1B). However, it appears that the addition of acetyl or tigloyl groups to the 3′- or 4′-carbon residues of *trans*-khellatone is required for cytoprotective function against β23 because *trans*-khellatone itself had no effect on β23-induced cytotoxicity in SH-SY5Y cells (Figure 1B). Further cell viability analysis revealed dose-dependent cytoprotective functions exerted by both PCiii and PCiv (Figure 1C). The median effective concentrations for PCiv and PCiii were 0.204 uM and 0.318 uM, respectively, indicating the slightly more potent cytoprotective function of PCiv. Notably, there was no obvious cytotoxicity even after treatment with high concentrations of PCiii and PCiv (up to 4 µM) (Figure 1C). As a mechanism of cytoprotection against amyloid mimic β23, PCiii facilitates proteasomal degradation of β23 [5]. Similarly, steady-state β23 expression levels were markedly reduced in SH-SY5Y cells treated with PCiv (Figure 1D,E). The degree of β23 reductions by PCiv was greater than that by PCiii (Figure 1D,E). This facilitation of β23 clearance by PCiii and PCiv was confirmed by immunofluorescence imaging of remnant HA-tagged β23 in SH-SY5Y cells (Figure 1F,G). While there was a persistent HA-β23 signal in mCherry reporter-expressing transfected cells in the DMSO-treated control group, the HA-β23 signal was markedly reduced in mCherry-positive transfected cells with PCiii and PCiv treatment (Figure 1F,G). Together, these results showed that the structural isomer PCiv is as effective as PCiii in suppressing amyloid-induced cytotoxicity through a mechanism similar to amyloid clearance.

### 2.2. PCiv Inhibits In Vitro α-Synuclein Aggregation and Prevents PFF-Induced α-Synucleinopathy in Neuron Cultures

**Figure 2:**
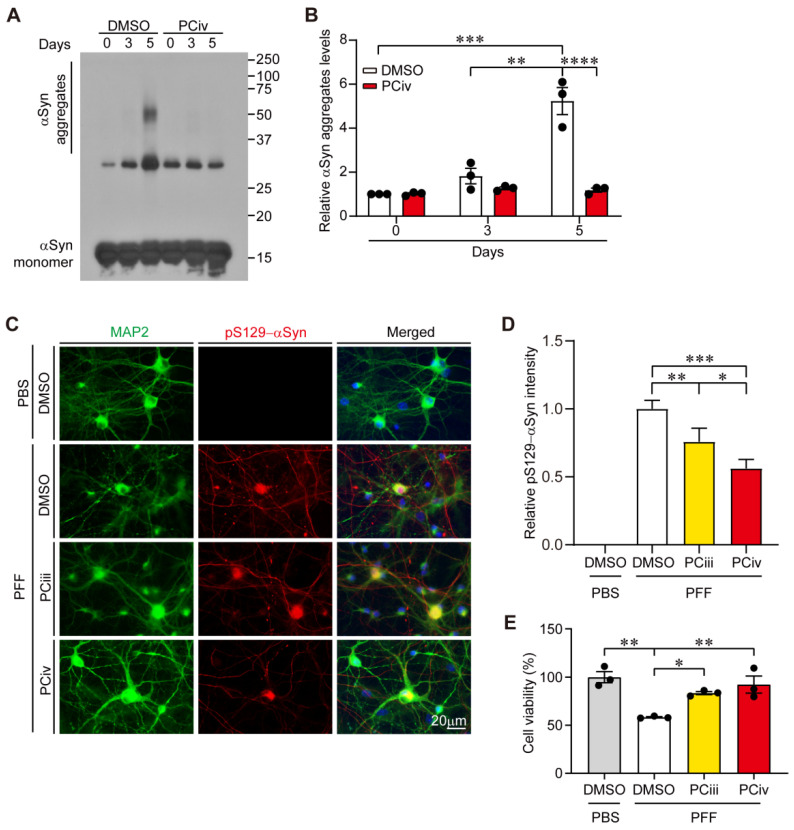
PCiv prevents α-synuclein aggregation in vitro and in cultured neurons with PFF treatment. (**A**) Representative Western blots showing the degree of recombinant α-synuclein (αSyn) aggregation in a test tube in the presence or absence of PCiv (100 μM) at the indicated incubation duration (phosphate-buffered saline [PBS], 37 °C for 3 or 5 days). Aggregation of α-synuclein was determined using α-synuclein antibody. (**B**) Quantification of the relative amount of α-synuclein aggregate in high molecular weight sizes in panel A (*n* = 3 per group). (**C**) Representative immunofluorescence of MAP2 and Lewy-like inclusion marker pS129-αSyn in mice cortical neurons treated with α-synuclein preformed fibril (PFF) (1 μg, for 14 days). To evaluate the protective effects of PCiv as compared with PCiii, these compounds were treated (1 μM every 2 days for 14 days) following the PFF treatment. (**D**) Quantification of pS129-αSyn immunofluorescence signal intensities in MAP2-positive cortical neurons in each experimental group (*n* = 15 cells from three separate experiments). (**E**) Cell viability assessment in the indicated experimental groups was monitored by CCK-8 assay (*n* = 3 per group). The data are expressed as means ± SEMs. * *p* < 0.05, ** *p* < 0.01, *** *p* < 0.001, and **** *p* < 0.0001, ANOVA test followed by Tukey’s post hoc analysis.

We previously reported that PCiii prevents PD-associated disease protein α-synuclein aggregation and toxicity through direct interactions with α-synuclein aggregates [5]. Since PCiv showed a more potent biological effect on cytoprotection and β23 clearance, we sought to determine the potential therapeutic function of PCiv against α-synuclein aggregation. Similar to PCiii, aggregation of recombinant α-synuclein into high molecular weight species was robustly blocked in the presence of PCiv, as monitored by Western blotting using α-synuclein-specific antibody (Figure 2A,B). When the biological effects on α-synuclein aggregation were compared, PCiii and PCiv presented a similar extent of inhibitory function against in vitro α-synuclein aggregation (Supplementary Figure S2A,B). The *t*-Khellatone, however, failed to prevent α-synuclein aggregation process (Supplementary Figure S2A,B). The inhibitory effect of PCiv on α-synuclein aggregation was further monitored in a cellular model of PD in primary cortical neuron cultures. For primary cultured mice cortical neurons, Lewy body-like inclusions were successfully induced 14 days following treatment with in vitro-prepared α-synuclein PFF. Consistent with these reports, PFF treatment of mice cortical neurons produced a marked elevation of pS129-α-synuclein (αSyn) and filament conformation--αSyn immunofluorescence signals (Figure 2C,D, and Supplementary Figure S2C,D), well-known markers for Lewy-like inclusions [8,9,10]. PCiv treatment mitigated both PFF-induced elevation of pS129-αSyn, and filament conformation--αSyn signals to a greater extent as compared to PCiii in PFF-treated cortical neurons (Figure 2C,D, and Supplementary Figure S2C,D). However, endogenous α-synuclein expression in either PBS- or PFF-treated mouse cortical neurons were not affected by PCiii or PCiv treatment (Supplementary Figure S2E,F). Supporting the notion that α-synuclein aggregation is neurotoxic, PFF treatment caused a substantial reduction of neuron viability up to approximately 50% compared to PBS control (Figure 2E). Both PCiii and PCiv treatment prevented this PFF-induced neurotoxicity with PCiv of slightly better neuroprotection (Figure 2E). Collectively, these results indicate that PCiv is highly effective in suppressing α-synuclein aggregation in vitro and in PFF-induced neuronal α-synucleinopathy, suggesting its potential therapeutic value in PD with Lewy pathologies.

### 2.3. Pharmacokinetic Analysis and Tissue Distribution of PCiv

**Figure 3:**
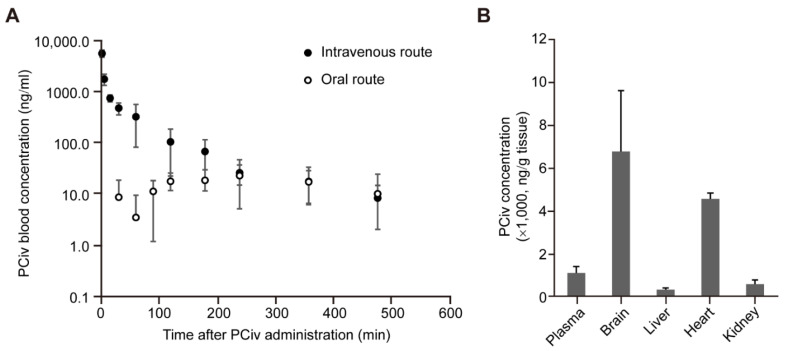
Pharmacokinetic analysis and tissue distribution of PCiv. (**A**) Pharmacokinetic analysis of blood PCiv levels at each indicated time point following intravenous or oral administration of PCiv (10 mg/kg) determined by ultra-performance liquid chromatography (*n* = 4 rats for oral and 5 rats for intravenous administration). (**B**) Quantification of PCiv levels in each indicated tissue (ng/g tissue weight) determined by ultra-performance liquid chromatography (*n* = 3 rats, tissues collected 15 min after intravenous PCiv administration (10 mg/kg). PCiv was dissolved in a vehicle (DMSO:PEG400:saline ratio of 5:70:25) for solubilization. The quantified data are expressed as means ± standard deviations.

We next sought to evaluate the pharmacokinetic profiles of PCiv to determine whether this compound would be adequate for in vivo preclinical applications. The plasma concentration-time profiles of intravenous and oral routes of administration in rats are presented as scatter plots with standard deviations (Figure 3A). The plasma PCiv concentration levels rapidly declined in a biphasic manner after intravenous bolus injection, whereas the plasma PCiv levels were observed to be relatively low after oral administration (Figure 3A). The calculated pharmacokinetic parameters are summarized in Supplementary Table S1. The mean systemic total clearance was 136.5 mL/min/kg with a short mean half-life (85.8 min), and the mean steady-state volume of distribution was 6620.9 mL/kg, indicating moderate-to-high tissue distribution of PCiv in rats. In addition, the oral pharmacokinetic properties of PCiv appear to be poor with low maximum concentration and absolute bioavailability (9.96%) (Supplementary Table S1). However, the tissue distribution in this study demonstrated that PCiv was efficiently distributed to the brain, the therapeutic target tissue (tissue- to-plasma concentration ratio of 6.4), whereas other major organs such as the liver and kidneys were found to have poor distribution characteristics with a low tissue-to-plasma concentration ratio (Figure 3B and Supplementary Table S2). Collectively, although PCiv was rapidly eliminated with poor oral absorption, the efficient brain penetration of PCiv in rats suggests that PCiv is highly permeable to the blood–brain barrier and can achieve therapeutic concentration levels in the target tissue (brain) in vivo.

### 2.4. Evaluation of PCiv Therapeutic Efficacy in a Sporadic PD Mouse Model of α-Synucleinopathy

The inhibitory biological activity of PCiv on α-synuclein aggregation and its favorable brain distribution profile prompted us to investigate the therapeutic effect of PCiv in a preclinical PD animal model. Combinatorial nigral injections of αSyn PFF and recombinant adeno-associated virus expressing α-synuclein (rAAV-αSyn) have been shown to recapitulate key pathological features of PD, including dopaminergic neuronal loss and Lewy-like inclusion formation as early as 1 month, contributing to motor dysfunction [11,12,13]. We coinjected PFF and rAAV-αSyn virus into the ventral tegmental area and substantia nigra pars compacta, respectively, to recapitulate α-synucleinopathy in mice (Supplementary Figure S3A). Mice were fed with a PCiv-containing diet (50 mg per kg diet) for the following 30 days to examine the potential biological and therapeutic effect of this compound in vivo (Supplementary Figure S3A). Each experimental mouse group displayed relatively similar spontaneous exploratory activity with no signs of anxiety in the open field test (Supplementary Figure S2B–D), although PD mice fed a regular diet exhibited a trend toward preferred exploration in the border zone.

**Figure 4:**
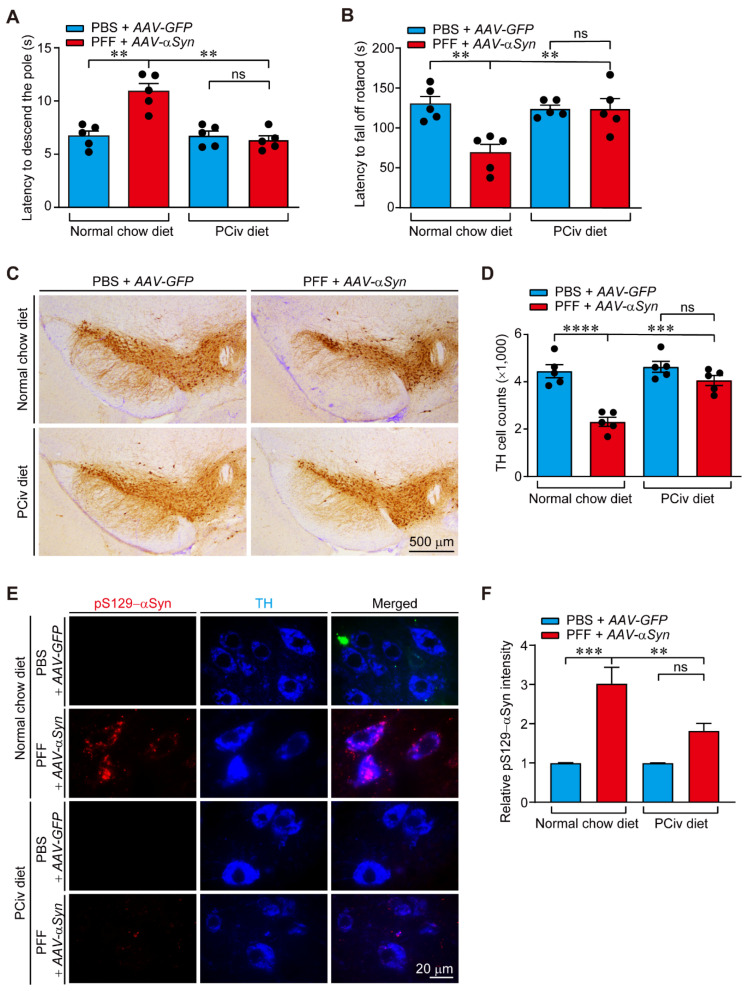
PCiv administration suppresses PFF-induced dopaminergic neurodegeneration, α-synuclein aggregation, and motor dysfunction in vivo. (**A**) Bradykinesia (slowness of movement) assessed by pole test for control and combinatorial PFF/*AAV-αSyn*-injected mice with or without PCiv treatment (50 mg PCiv/kg diet for 30 days, *n* = 5 per group). Control mice are those nigrally injected with PBS and *AAV-GFP*. (**B**) Motor coordination of the mice in the experimental group was assessed by an accelerated rotarod test (*n* = 5 per group). (**C**) Representative tyrosine hydroxylase (TH) immunohistochemical staining of the ventral midbrains of control and combinatorial α-synucleinopathy-PD mice fed with a PCiv diet (50 mg/kg diet for 30 days) or normal chow diet. (**D**) Stereological assessment of TH-positive dopaminergic neurons in the substantia nigra pars compacta of the injection side of the indicated mice groups (*n* = 5 mice per group). (**E**) Representative immunofluorescence images of pS129-αSyn and TH in the substantia nigra coronal sections of the indicated experimental mice groups. (**F**) Quantification of relative pS129-αSyn fluorescence intensities in the TH-positive dopamine neurons of the substantia nigra sections from the indicated experimental groups (*n* = 28 cells from 3 mice per group). The quantified data are expressed as means ± SEMs. Statistical significance was determined by an ANOVA test with Tukey’s post hoc analysis, ** *p* < 0.01, *** *p* < 0.001, and **** *p* < 0.0001. ns = nonsignificant.

This sporadic PD mouse model with combinatorial PFF and rAAV-αSyn nigral injections under a normal diet manifested motor dysfunctions as monitored by the pole (for bradykinesia) and rotarod (for motor coordination) tests (Figure 4A,B). This development of motor impairment in PD mouse models was almost completely prevented in mice fed with a diet containing PCiv for four weeks (Figure 4A,B). Consistent with this striking rescue effect of PCiv on PD-associated behavioral deficits, we observed marked protection of dopaminergic neurons in the combinatorial-injected PD mouse model under a PCiv diet (Figure 4C,D). The extent of dopamine neuron survival in the PCiv-treated PD mouse model was comparable to that in control mice receiving regular or PCiv diets (Figure 4C,D). Importantly, PCiv treatment in the control mouse group had no effect on dopamine neuron viability (Figure 4C,D), suggesting that PCiv itself does not induce neurotoxicity in dopamine neurons.

Dopaminergic α-synuclein aggregation pathology was monitored by co-immunolabeling of tyrosine hydroxylase (TH)-positive-dopamine neurons with the Lewy-like inclusion marker, pS129-αSyn, in coronal ventral midbrain sections from each experimental mouse group. Combinatorial PFF and rAAV-αSyn nigral injection led to the robust formation of Lewy-like inclusions in TH-positive dopamine neurons (Figure 4E,F). This α-synuclein aggregation pathology was largely blocked by PCiv in the combinatorial α-synucleinopathy PD mouse model (Figure 4E,F). There was no alteration of total α-synuclein expression by PCiv administration in both PBS+AAV-GFP injection and PFF+AAV-αSyn injection groups (Supplementary Figure S4A,B). Taken together, our preclinical evaluation of PCiv in a PD mouse model indicated the potential translational value of PCiv in disease-modifying therapy for PD with α-synuclein pathology.

## 3. Discussion

Here, we are the first to report the therapeutic biological function of PCiv in preventing α-synuclein aggregation and neurotoxicity in an animal model of PD. In our previous report characterizing PCiii, which was initially screened as a natural anti-amyloid compound [5], PCiv was observed as merely a byproduct of higher synthetic yield without further evaluation of its potential biological activity. PCiv and PCiii are structural isomers differing in the positions of acetyl or tigloyl conjugation at the 3′- or 4′-carbon residues of *trans*-khellactone. Attachment of the tigloyl moiety at the 3′-carbon of *trans*-khellactone seems to provide slightly better cytoprotection against amyloid-induced cytotoxicity and a greater protective effect on PFF-induced α-synuclein aggregation in neurons. PCiv has never been studied for its potential inhibitory effect against α-synuclein aggregation, although there are some reports on the biological activity of the peucedanocoumarin analog modulating platelet aggregation [14]. The comparable inhibitory effect of both PCiii and PCiv on recombinant α-synuclein protein aggregation in vitro suggests that the arrangement of acetyl or tigloyl moieties on *trans*-khellactone is not critical for α-synuclein interaction and inhibition of its aggregation. Although the actual binding mode of PCiii or PCiv to α-synuclein aggregates is unclear, this result may suggest the potential presence of multiple binding pockets on α-synuclein aggregate structures or that there is some flexibility on the 3′- and 4′-C side of peucedanocoumarins for α-synuclein interaction. However, it is evident that acetyl and tigloyl conjugation is critically important for the biological activity of PCiii and PCiv since *trans*-khellatone had no effect on cytoprotection against β23-induced cellular toxicity. It would be instructive to synthesize a more extensive repertoire of structural derivatives of PCiii or PCiv with different moieties conjugated to 3′ and 4′-C and evaluate their anti-protein aggregate biological activity. Moreover, it requires further investigation to understand which stereoisomer of racemic PCiii or PCiv mixture exerted actual biological effect on α-synuclein aggregation process. It would also be informative to narrow down which domain of α-synuclein aggregates PCiii and PCiv physically interacts during the disaggregation process. Hydrogen deuterium exchange mass spectrometry may be an excellent approach for investigating this issue in the future.

In this study, the therapeutic efficacy of PCiv was evaluated in sporadic PD mouse models. Although PCiii was reportedly neuroprotective in a 6-OHDA-induced PD mouse model [6], PCiv showed more potent prevention of PFF-induced α-synuclein aggregation in mice cortical neurons (Figure 2C), prompting us to further examine its therapeutic potential in a PD mouse model of α-synucleinopathy. With favorable brain penetration of PCiv in vivo, PCiv treatment prevented α-synuclein aggregation and dopaminergic neurodegeneration in a PD mouse model with a combinatorial PFF and *AAV-αSyn* nigral injection, ultimately contributing to the behavioral rescue of motor functions. α-Synuclein aggregation pathologies are characterized by progressive impairment of brain function mediated by various brain subregions. According to Braak’s theory, which was supported by experimental data [4,9,15], α-synuclein aggregation can be initiated in the intestinal peripheral nervous system or olfactory nerve; this Lewy pathology can then propagate to higher brain structures in the central nervous system through retrograde transmission. Indeed, targeting α-synuclein aggregation could be beneficial for terminating Lewy pathology propagation at any Braak stages. Furthermore, PCiv-mediated prevention of α-synuclein aggregation can preserve motor and non-motor functions mediated by specific brain subregions, even in environments with Lewy pathology infiltration. PCiv may be further evaluated in other α-synuclein transgenic mouse models, affecting multiple brain regions, to expand its therapeutic application to more diverse neurological disorders. Interestingly, the PCiv analog was reported as a potential probe in a cell model of huntingtin aggregation according to the PubChem Bioassay (CHEMBL1378488). PCiii was shown to be effective as an anti-aggregate compound for both α-synuclein and huntingtin proteins. Similarly, PCiv likely retains anti-aggregate functions that are applicable to a broader spectrum of disease-associated protein aggregation. Amyloid-mimic β23 clearance facilitated by PCiv supports this notion.

The advantages of PCiv compared to PCiii include a high yield in organic synthesis and a more favorable pharmacokinetic profile. Previously, we failed to analyze the detailed pharmacokinetic profiles of PCiii likely due to the poor pharmacokinetic properties, although it demonstrated brain distribution following intraperitoneal administration in mice [6]. In contrast, PCiv was well-dissolved in the prepared vehicle solution (DMSO:PEG400:saline ratio of 5:70:25), and we were able to obtain pharmacokinetic profiles of PCiv following oral administration in rats. Although PCiv has approximately a 1.5-h half-life and relatively low bioavailability of 10%, its efficient distribution to the target organs (6.4-fold compared with the plasma concentration) poses strong advantages for PCiv in clinical applications for PD with oral administration. The pharmaceutical preparation of PCiv to enhance its oral bioavailability and absorption may be implemented for lead compound optimization. Moreover, toxicity evaluation of PCiv is critically important prior to the clinical evaluation of this drug. Although we did not observe any overt toxicity in cells and in vivo mouse models, prolonged and high-dose PCiv treatment in mice and characterization of behavior and organ pathology are required to ensure the safety of the drug, especially when PD treatment is likely to be prolonged because of the chronic nature of the disease.

## 4. Materials and Methods

### 4.1. Chemicals and Antibodies

Goat serum (#01-6201) and doxycycline (#D9891) were purchased from Invitrogen, Thermo Fisher Scientific Inc., Waltham, MA, USA and Sigma-Aldrich, St. Louis, MO, USA, respectively. The following primary antibodies were used: mouse antibody to HA (#PA1-985, 1:5000, Roche, Basel, Switzerland), rabbit antibody to filament α-synuclein (#ab209538, 1:5000, Abcam, Cambridge, UK), rabbit antibody to MAP2 (#ab32454, 1:1000, Abcam, Cambridge, UK), mouse antibody to α-synuclein (#610787, 1:3000, BD transduction laboratories, San Jose, CA, USA), mouse antibody to pS129-α-synuclein (#825702, 1:3000, Biolegend, San Diego, CA, USA), and rabbit antibody to TH (#NB300-109, 1:2000, Novus Biologicals, Centennial, CO, USA). The following secondary antibodies were used: horseradish peroxidase (HRP)-conjugated goat antibody to mouse immunoglobulin (Ig)G (#Gtx-213111-01, 1:5000, Genetex, Irvine, CA, USA), HRP-conjugated goat antibody to rabbit IgG (#Gtx-213110-01, 1:5000, Genetex, Irvine, CA, USA), biotin-conjugated goat antibody to rabbit IgG (#BA-1000, 1:1000, Vector Laboratories, Burlingame, CA, USA), Alexa Fluor 488-conjugated goat antibody to mouse IgG (A21202, 1:1000; Invitrogen, Thermo Fisher Scientific Inc., Waltham, MA, USA), Alexa Fluor 568-conjugated donkey antibody to rabbit IgG (#A10042, 1:1000; Invitrogen), and HRP-conjugated mouse antibody to β-actin (cat# A3854, 1:10,000, Sigma-Aldrich, St. Louis, MO, USA).

### 4.2. Organic Synthesis of PCiv

The general procedure was performed as follows. Unless otherwise noted, all starting materials and reagents were obtained from commercial suppliers and used without further purification. All solvents used for the routine isolation of products and chromatography were of reagent grade and glass-distilled. Reaction flasks were dried at 100 °C. Air- and moisture-sensitive reactions were performed in an argon atmosphere. Chemical shifts are expressed in parts per million (*δ*) downfield from tetramethylsilane and referenced to deuterated solvent (CHCl_3_). ^1^H-NMR data were reported in the order of chemical shift, multiplicity (s, singlet; d, doublet; t, triplet; q, quartet; m, multiplet or multiple resonances), number of protons, and coupling constant in hertz (Hz).

To a solution of **2** (340 mg, 1.0 mmol) in CH_2_Cl_2_ (10 mL), *i*Pr_2_NEt (0.35 mL, 2.0 mmol) and acetic anhydride (0.14 mL, 1.5 mmol) were added at 0 °C. The reaction mixture was then warmed to room temperature and stirred for 12 h. After aq.NH_4_Cl was added to the reaction mixture, it was transferred to a separatory funnel and extracted with CH_2_Cl_2_ twice. The organic layers were dried over MgSO_4_, filtered, and evaporated *in vacuo*. The residue was purified by column chromatography on silica gel (ethyl acetate: *n*-hexane = 1:2) to afford PCiv **3** (320 mg, 83%) as an amorphous solid.

PCiv **3**: ^1^H-NMR (CDCl_3_, 500 MHz) δ 7.58 (1H, d, *J* = 15.6 HZ), 7.35 (1H, d, *J* = 14.5 Hz), 6.80 (1H, m,), 6.78 (1H, d, *J* = 14.5 Hz), 6.24 (1H, d, *J* = 7.5 Hz), 6.21 (1H, d, *J* = 15.6 Hz), 5.31 (1H, d, *J* = 8.0 Hz), 2.08 (3H, s), 1.78 (3H, t, *J* = 1.0 Hz), 1.74 (3H, dd, *J* = 12.0, 1.5 Hz), 1.41 (3H, s), 1.33 (3H, s); ^13^C-NMR (CDCl_3_, 125 MHz) δ 169.5, 166.3, 159.9, 156.5, 154.0, 143.3, 138.9, 129.1, 127.7, 114.4, 113.1, 112.5, 107.0, 71.9, 63.8, 24.2, 22.6, 20.7, 14.5, 12.1. LRMS (FAB) *m*/*z* 386 (M^+^); HRMS (FAB) Calcd for C_21_H_22_O_7_ (M^+^): 386.1366, Found 386.1381.

### 4.3. Cell Culture and Transfection

Human neuroblastoma SH-SY5Y cells (ATCC, Manassas, VA, USA) were grown in complete media containing DMEMs, 10% fetal bovine serum (vol/vol), and antibiotics (penicillin-streptomycin 100 U/mL, Thermo Fisher Scientific, Waltham, MA, USA). The atmospheric condition used to preserve the cells consisted of 5% CO_2_/95% air at 37 °C and maintained a humidified environment. X-tremeGENE HP transfection reagents (Roche, Basel, Switzerland) were used for transient transfection of DNA constructs (pCMV-tTA, TetP-β23, and pTet-dual2 [5]) following the manufacturer’s instructions.

### 4.4. Cell Viability Assay

SH-SY5Y cells were plated in 12-well plates at a density of 0.2 × 10^6^ cells per well. Following transient transfection with the indicated constructs and subsequent treatment protocol, the cells were harvested by trypsinization, thereby yielding single-cell suspensions. The cells were washed twice with phosphate-buffered saline (PBS) and resuspended in serum-free DMEM. The resuspended cells were mixed with an equal volume of 0.4% trypan blue (*w*/*v*) and incubated for 2 min at room temperature. Live and dead cells were counted using a Countess II Automated Cell Counter (Life Technologies, Carlsbad, CA, USA) for assessing cell viability.

### 4.5. Fluorescence Imaging

The SH-SY5Y cells were fixed with 4% paraformaldehyde in PBS and blocked with a blocking solution (5% normal goat serum [Invitrogen], 0.1% Triton X-100 [Sigma-Aldrich, St. Louis, MO, USA]) in PBS, pH 7.4) for 1 h at room temperature and incubated with primary antibodies against HA-tagged β23 at 4 °C overnight. Then, the cells were washed with wash buffer (0.1% Triton x-100 in PBS, pH 7.4) and incubated with corresponding secondary antibodies conjugated with fluorescent dyes at room temperature for 1 h. After simple washing with wash buffer, the samples were incubated with DAPI (Sigma-Aldrich, St. Louis, MO, USA) solution. The cells were washed with DAPI solution in PBS and mounted on a microscope slide using Immu-Mount solution (Thermo Fisher Scientific, #9990402). Images were obtained using a fluorescent microscope (Axio Imager M2; Carl Zeiss, Oberkochen, Germany).

### 4.6. Western Blotting

Lysis buffer was added to the SH-SY5Y cells washed with ice-cold PBS (1% Nonidet P40 in PBS, pH 7.4) that was supplemented with protease/phosphatase inhibitors. To obtain total protein lysate, we performed freezing and thawing cycles three times using dry ice and ice water. The samples were then centrifuged at 14,000 g for 30 min. The supernatants were quantified through BCA protein assessment. The samples were mixed with 2X Laemmli buffer (Bio-Rad, Hercules, CA, USA) supplemented with β-mercaptoethanol (Sigma-Aldrich) and boiled for 5 min at 95 °C. The proteins were separated by SDS-PAGE and transferred to nitrocellulose membranes for immunoblotting. Western blotting was performed using specific antibodies and the immune response bands were visualized using chemiluminescence (Pierce, Thermo Fisher Scientific, Waltham, MA, USA). Next, band quantification was performed using ImageJ software. (National Institutes of Health, Bethesda, MD, USA).

### 4.7. α-Synuclein Aggregation Assessment and PFF Preparation

Plasmid pRK172-human α-synuclein was transformed into BL21-competent *Escherichia coli* using heat-shock transformation. Recombinant α-synuclein was induced and purified, as described above. Purified α-synuclein was incubated with or without PCiv in sodium acetate buffer (100 mM, pH 7.5) in a shaking incubator (300 rpm, Vision Scientific, Daejeon, Korea) at 37 °C. After the indicated days of incubation, fibril formation in the protein solution was assessed by Western blotting. α-Synuclein fibrils (5 mg/mL) generated from monomeric α-synuclein incubation for 7 days were processed by sonication (Vibra Cell, Sonics & Materials, Inc., Newtown, CT, USA) to prepare α-synuclein PFFs. To prevent the solution from overheating, 60 pulses with a 0.5 s duration of 10% of the power level were applied with a short pause after every 10 pulses.

### 4.8. Mouse Cortical Neuron Culture and PFF Treatment and Neurotoxicity Assessment

CD1 mice were purchased from Orient Co. for use in the mouse primary neuron culture. E14 embryos from CD1 mice were dissected for primary cortical neurons in Neurobasal A medium (Gibco, Thermo Fisher Scientific Inc., Waltham, MA, USA) supplemented with B-27 (Gibco, Thermo Fisher Scientific Inc., Waltham, MA, USA) and 1 mM L-glutamine. Next, the primary cortical neurons were maintained in a poly L-lysine-coated tissue culture plate, and the medium was changed every 2–3 days. After 7 days, PFF was added to the cortical neurons and incubated for 14 days to observe the p-S129-α-synuclein signal which was used for immunofluorescence analysis. For neurotoxicity assessment, mouse cortical neurons following 14 days of PFF treatment were incubated with Neurobasal A medium containing CCk-8 (cat# B1007-500, Cellrix, MediFab, Seoul, Korea) solutions for 1.5 h in 37 ℃. The optical value was read at 450 nm using a multi-detection microplate reader (Synergy, BioTek Instruments Inc., Winooski, VT, USA).

### 4.9. Animal Experiments

All animal experiments were approved by the Ethical Committee of Sungkyunkwan University Animal Care and Use Committee of Gachon University (Approval number: SKKUIACUC2019-06-15-1; GIACUC-R2021006) and were conducted in accordance with all applicable international guidelines. Male C57BL/6N mice (2 months old) were obtained from Orient Bio (Suwon, Korea). The animals were housed in air-controlled rooms with a 12-h dark/light cycle. Mice and rats were provided ad libitum access to diet and water. All efforts were made to minimize animal suffering and the number of animals used in the experiments. A synthetic PCiv diet was supplied to the mice. The PCiv diet administration (50 mg/kg/day for PFF PD mouse models) began on day 0 and was continued for 30 days. Intranigral injection of the *rAAV-αSyn* and PFF mixture was performed on day 0. Mice brains were prepared for analysis, as described below. To test the therapeutic effects of PCiv, we prepared a diet containing PCiv. To facilitate each mouse consuming 2 mg of PCiv per day, we generated a mixture of 400 mg of PCiv per kg of rodent diet and then supplied it to the mice.

### 4.10. Rat Pharmacokinetic and Tissue Distribution of PCiv

In vivo rat pharmacokinetic studies were performed for intravenous and oral administration of PCiv as previously reported [16,17,18]. Sprague-Dawley rats (8 weeks old, 260–280 g) were purchased from Orient Bio Inc. (Seongnam, Korea). Rats were maintained with free access to food and water under a 12:12 h light/dark cycle for 1 week. For pharmacokinetic studies, under anesthetic conditions (20 mg/kg tiletamine/zolazepam [Zoletil; Vibrac Laboratories, Carros, France] administered intramuscularly), the femoral vein and artery were surgically cannulated using polyethylene tubing (PE50; Clay Adams, Parsippany, NJ, USA) in fasted rats. Then, PCiv was administered intravenously and orally at a dose of 10 mg/kg (n = 4–5). The drug was dissolved in a vehicle (DMSO:PEG400:saline at a ratio of 5:70:25). Blood samples (approximately 100 μL) were collected at predetermined time points (0, 1, 5 (intravenous administration only), 15, 30, 60, 120, 180, 240, 360, and 480 min) after drug administration. To prevent blood clots and fluid loss, the cannula to the femoral artery was flushed with 20 IU/mL heparinized saline, and the same volume of saline was pushed to the cannula for the femoral vein. The plasma fraction was immediately obtained after centrifugation at 14,000 rpm for 15 min at 4 °C and then stored at −20 °C prior to analysis using the ultra-high-performance liquid chromatography (UHPLC)-diode array detector (DAD) method. Similarly, a tissue distribution study was performed 15 min after the intravenous bolus injection (10 mg/kg), as previously described [17,18]. Four major tissues, including tissues from the brain, heart, kidneys, and liver, were collected. Based on the wet weights of the tissue samples, a two-fold volume of PBS was added and homogenized. The tissue homogenates were stored at −20 °C before UHPLC-DAD analysis. The UHPLC-DAD system consisted of an Agilent Technologies 1290 Infinity II equipped with a DAD (G7117A), an autosampler (G71678B), a binary pump (G7104A), and a multicolumn thermostat (G7116B). The PCiv level was determined using a CapcellpakTM C18 5 μm column (250 × 1.5 mm, Shiseido Fine Chemical Co., Tokyo, Japan), as previously described [6]. The mobile phase was composed of 0.1% phosphoric acid and acetonitrile and eluted in the gradient mode at a flow rate of 0.2 mL/min. A DAD detector was applied at 321 nm, with an injection volume of 10 μL. The calibration range was 20–10,000 ng/mL with good linearity (R = 0.99783 with 1/× weighting). The tissue concentration levels were calculated as the PCiv amount (ng) per gram of organ weight.

### 4.11. Nigral Co-Injection of PFF and AAV-αSyn

The α-synucleinopathy PD model was created by co-injecting PFF (10 µg; ventral tegmental area: AP, −3.4 mm; ML, −0.5 mm; DV, −4.3 mm) and *rAAV-αSyn* (AAV serotype 1, 1 µL of titer 5 × 1011 GC/mL; substantia nigra pars compacta: AP, −3.4 mm; ML, −1.3 mm; DV, −4.3 mm) into a 2-month-old C57BL/6N mouse. *rAAV-GFP* was used as the control. PFF was prepared from pure recombinant human α-synuclein (Proteos, Inc., Kalamazoo, MI, USA) according to a previous report. Before use, sonicated PFFs were evaluated using Western blotting and a functional assay in cortical neuron cultures (pS129-αSyn induction and neurotoxicity). After co-injecting PFF and *rAAV-αSyn*, PD models were fed a PCiv or normal diet for a month. For stereological analysis, the animals were perfused 1 month after intranigral injection and fixed intracardially with ice-cold PBS. The tissues were post-fixed with 4% paraformaldehyde overnight. The fixed mice brains were cryoprotected in 30% sucrose (wt/vol) in PBS and subsequently processed for immunohistochemistry.

### 4.12. TH Stereological Cell Counting and Immunohistochemistry

Coronal sections (thickness, 35 µm) of the fixed and cryoprotected brains were cut, including the substantia nigra. The sections were incubated with rabbit polyclonal anti-TH (1:1000, Novus Biologicals, Centennial, CO, USA) antibody followed by sequential incubations with biotinylated goat anti-rabbit IgG and streptavidin-conjugated HRP using a Vectastain ABC kit (Vector Laboratories, Burlingame, CA, USA) according to the manufacturer’s instructions. To visualize TH-positive cells, 3,3-diaminobenzidine (DAB, cat# D4293, Sigma-Aldrich, St. Louis, MO, USA) was used as the HRP substrate. The total number of TH-positive neurons in the substantia nigra pars compacta was determined using the Optical Fractionator probe in Stereo Investigator software (MicroBrightfield, Williston, VT, USA). All stereological counting was performed in a manner blinded to the mouse treatments. For immunofluorescence labeling, coronal ventral midbrain sections were blocked in a blocking buffer (1× PBS, 5% normal goat serum, and 0.2% Triton-X) for 1 h at room temperature. The brain sections were incubated with the primary antibody mixture overnight at 4 °C, followed by brief washing and subsequent incubation in PBS containing fluorescent dye-labeled secondary antibodies. Fluorescent images were obtained using a fluorescence microscope (Axiovert, 200 M; Carl Zeiss, Oberkochen, Germany) for image acquisition.

### 4.13. Behavior Tests

#### 4.13.1. Open Field Test

The open field uses a square plastic box (40 cm × 40 cm × 40 cm) divided into 64 (8 × 8) equal areas (5 cm × 5 cm). The field was divided into border, peripheral, and central areas. The central area contained four central squares (2 × 2). The peripheral area contained 12 squares surrounding the central region, and the border area comprised the remaining squares. The mice were placed in the middle of the box and left for 15 min. After each test, 70% ethanol was used to clean the open fields. Every trial used Smart v3.0 software (Panlab, Barcelona, Spain) to record the distance traveled as a measure of locomotor activity and the time spent in and entries to the center as an anxiolytic indicator.

#### 4.13.2. Rotarod Test

Before the test trial, the mice were trained to walk in the rotarod for 2 days. On the test day (day 3), the mice were placed on the accelerating cylinder, and the latency and speed of falling off the rotarod were recorded. The speed of the rotarod was increased from 4 to 40 rpm within 5 min. The test was terminated when the mouse fell off the rotarod.

#### 4.13.3. Pole Test

The pole is a bandaged metal rod that is 58 cm long and 10 mm in diameter. For the pole test, mice were allowed to adapt to the cage for at least 5 min. When the mice were adapted to the cage with the pole, they were placed at the end of the stick facing down. The total time until the animal came down the pole and put its forefoot on the floor was recorded. Latency was calculated as the average time of the three trials.

### 4.14. Statistics

Quantitative data are presented as the mean ± standard error of the mean. Statistical significance was assessed using an analysis of variance test with Tukey’s honest significant difference post hoc analysis for comparisons of more than three groups. Differences were considered statistically significant at *p* < 0.05. GraphPad Prism software (GraphPad Software, San Diego, CA, USA) was used to prepare all plots and perform all statistical analyses.

## Data Availability

The data presented in this study are available in the article [and/or] its supplementary materials.

## Supplementary Materials

The following supporting information can be downloaded at: https://www.mdpi.com/article/10.3390/ijms23158618/s1.

## Abbreviations

αSyn	α-synuclein
AAV	adeno-associated virus
DAD	diode array detector
HRP	horseradish peroxidase
PBS	phosphate-buffered saline
PCiii	peucedanocoumarin III
PCiv	peucedanocoumarin IV
PD	Parkinson’s disease
PFF	preformed fibril
TH	tyrosine hydroxylase
UHPLC	ultra-high performance liquid chromatography
