# Supplementary material for: Synthetic Peucedanocoumarin IV Prevents α-Synuclein Neurotoxicity in an Animal Model of Parkinson’s Disease
Source: Int J Mol Sci. 2022 Aug 3;23(15):8618. doi: 10.3390/ijms23158618 (PMC9369442; doi:10.3390/ijms23158618)
Supplement: Supplementary file 1 [file ijms-23-08618-s001.zip › ijms-1771308-supplementary.pdf]

Supplementary figures

Synthetic peucedanocoumarin IV prevents α-synuclein neurotoxicity in an animal model of Parkinson's disease

Heejeong Kim, Han-Joo Maeng, Ji Hun Kim, Jin-Ha Yoon, Yohan Oh, Seung-Mann Paek, and Yunjong Lee

A

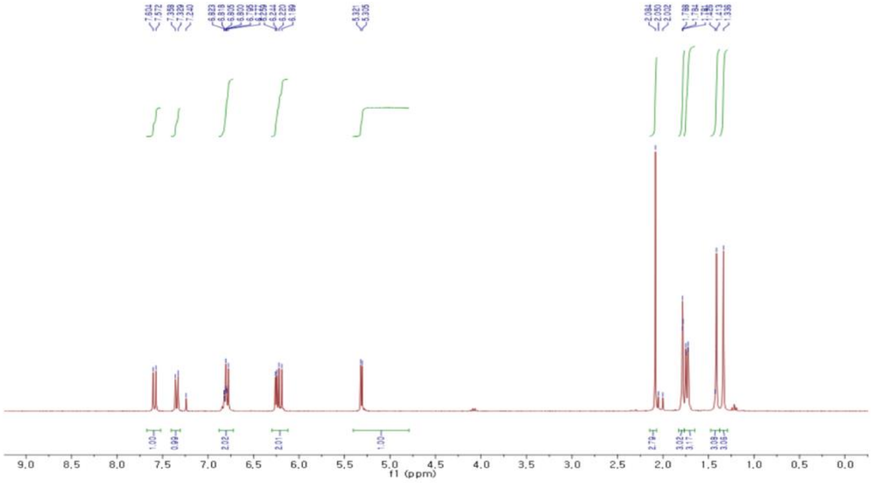

B

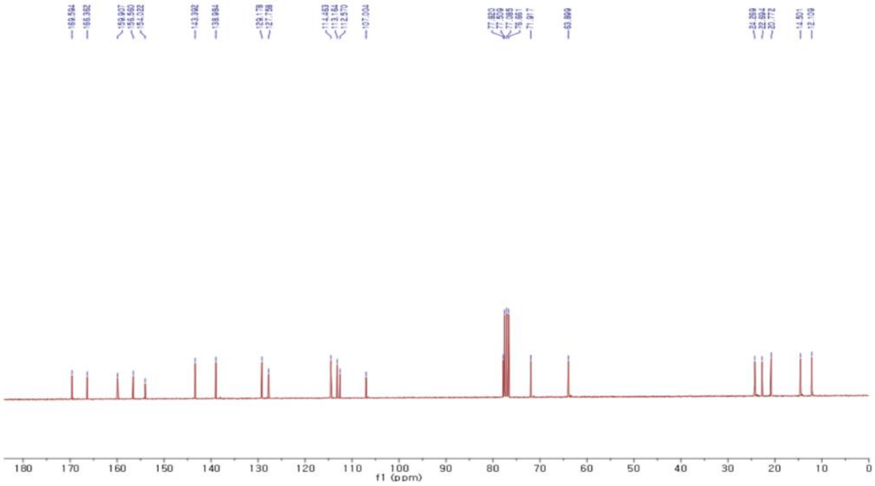

Supplementary Figure S1. NMR analysis of synthetic PCiv

(**A**) ^{1}H-NMR chart of synthesized PCiv.

(**B**) ^{13}C-NMR chart of synthesized PCiv.

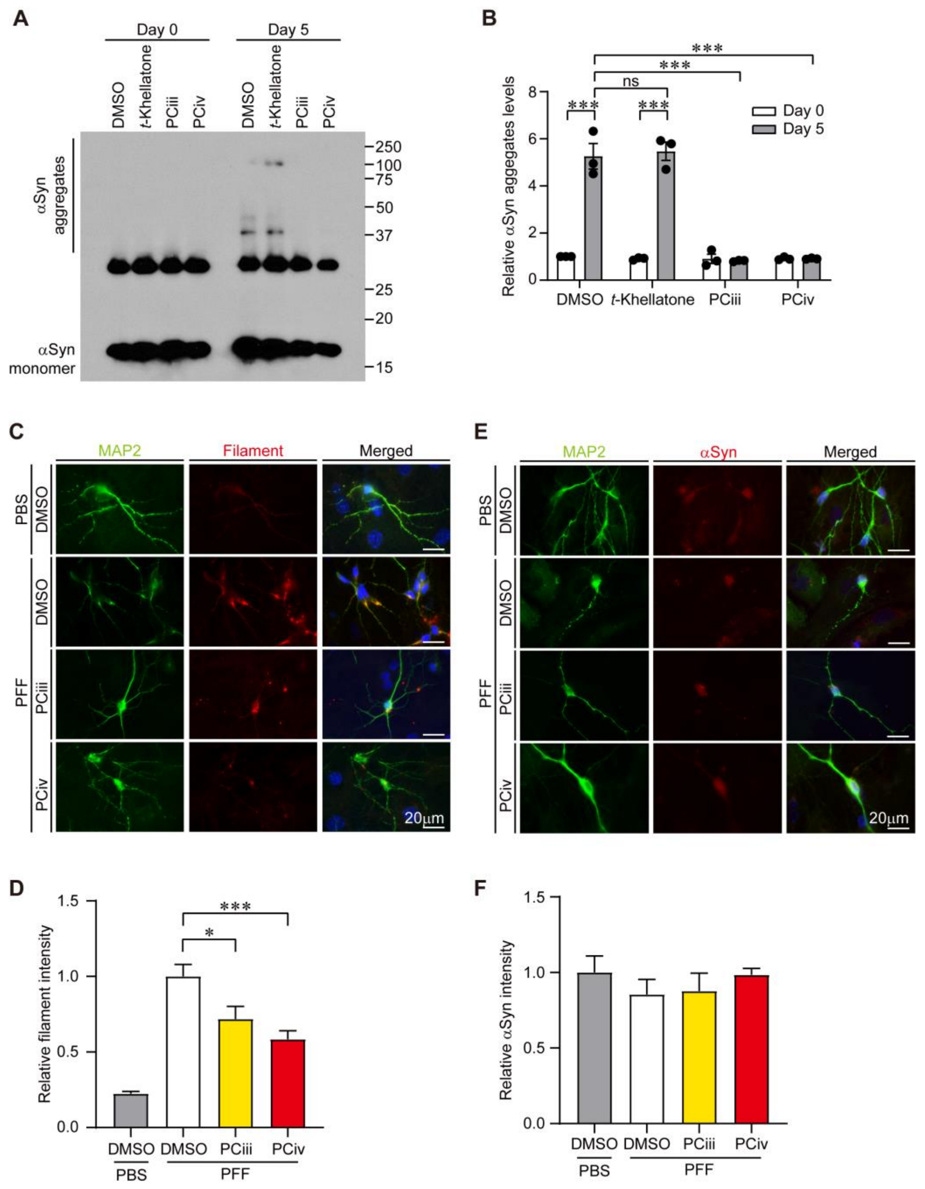

Supplementary Figure S2. PCiv prevents a-synuclein aggregation in vitro and in cell α-synucleinopathy model

(**A**) Representative Western blots showing the degree of recombinant α-synuclein (αSyn) aggregation in a test tube in the presence or absence of *t*-Khellatone, PCiii, and PCiv (100 μM) at 0 and 5 days of incubation (phosphate-buffered saline [PBS], 37 °C for 5 days). Aggregation of α-synuclein was determined using α-synuclein antibody. (**B**) Quantification of relative amount of α-synuclein aggregate in high molecular weight sizes in panel A (*n* = 3 per group). (**C**) Representative immunofluorescence of MAP2 and filament conformation-αSyn (Filament) in mice cortical neurons treated with α-synuclein preformed fibril (PFF) (1 μg, for 14 days) in the presence or absence of PCiii and PCiv (1 μM every 2 days for 14 days). (**D**) Quantification of filament conformation-αSyn (Filament) immunofluorescence signal intensities in MAP2-positive cortical neurons in each experimental group (*n* = 15 cells from three separate experiments). (**E**) Representative immunofluorescence of

MAP2 and αSyn in mice cortical neurons of the treatments applied in the panel (**C**). (**F**) Quantification of αSyn immunofluorescence signal intensities in MAP2-positive cortical neurons in each experimental group (n = 15 cells from three separate experiments). The data are expressed as means ± SEMs. *$P < 0.05$, ***$P < 0.001$, ANOVA test followed by Tukey's post hoc analysis. ns, non-significant.

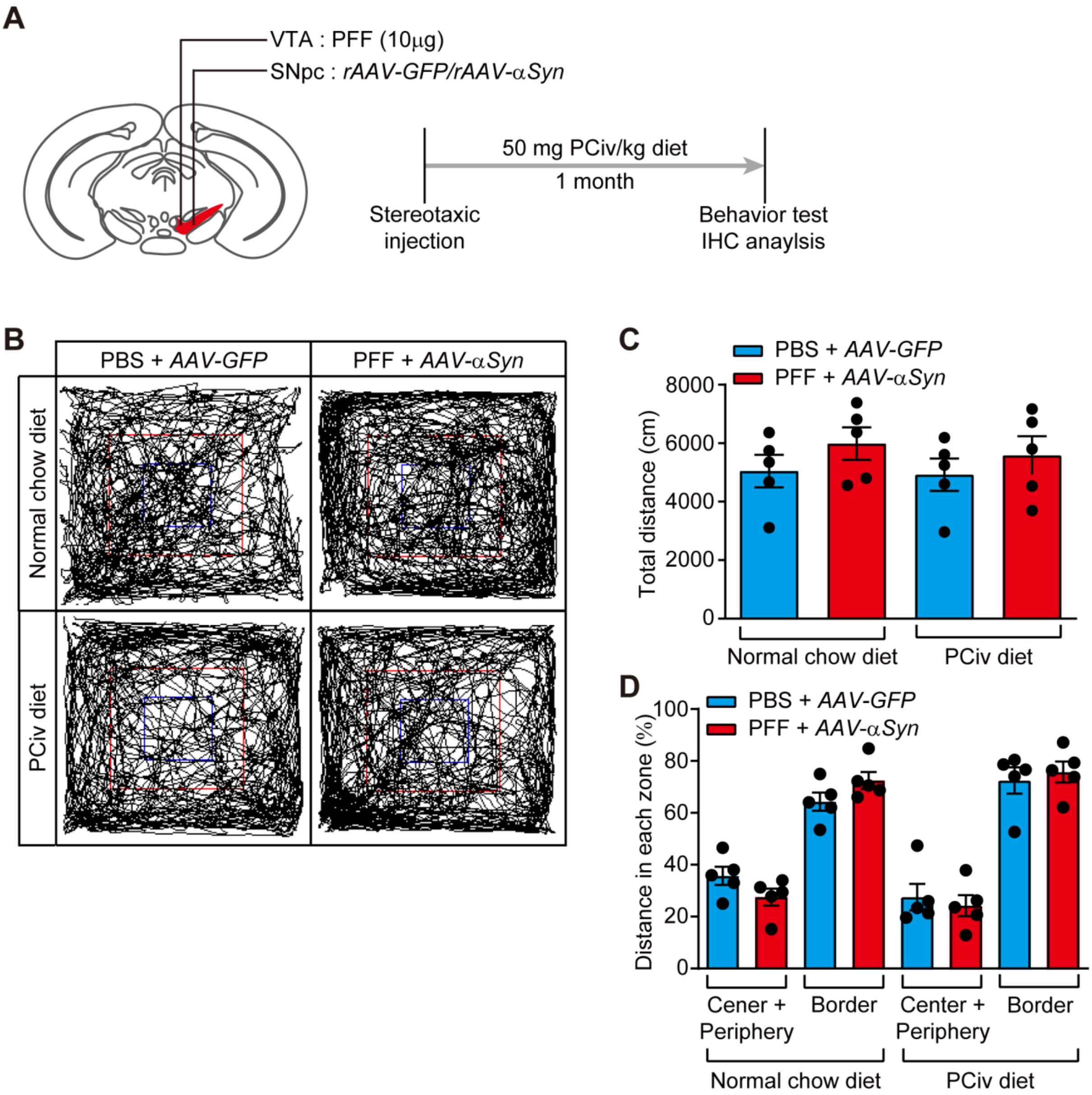

Supplementary Figure S3. Open field exploration of mice with PCiv administration

(**A**) Schematic summary of experimental procedure. PFF and rAAV-αSyn were injected into ventral tegmental area (VTA), and substantia nigra pars compacta (SNpc) subregions of one hemisphere of mice, respectively. PBS + rAAV-GFP coinjection group was used as control. PCiv diet (50 mg per kg diet for 30 d) was administered following PFF and rAAV-αSyn nigral injections and continued for 30 days until subsequent behavioral and pathological analysis.

(**B**) Representative exploratory paths of mice nigrally injected with PFF and AAV-αSyn and treated with PCiv (50 mg/kg diet) or a regular diet in an open-field test. The "Center + Periphery" zone is the area inside the red box, and the "Border" zone is the area outside of the red box.

(C) The total distance traveled by mice in each experimental group during the 15-min open field test session (*n* = 5 per group).

(C) Percentage of distance spent in the Center + Periphery or Border zones of the open field arena as an indicator of anxiety (n = 5 mice per group). The data are expressed as means ± SEMs.

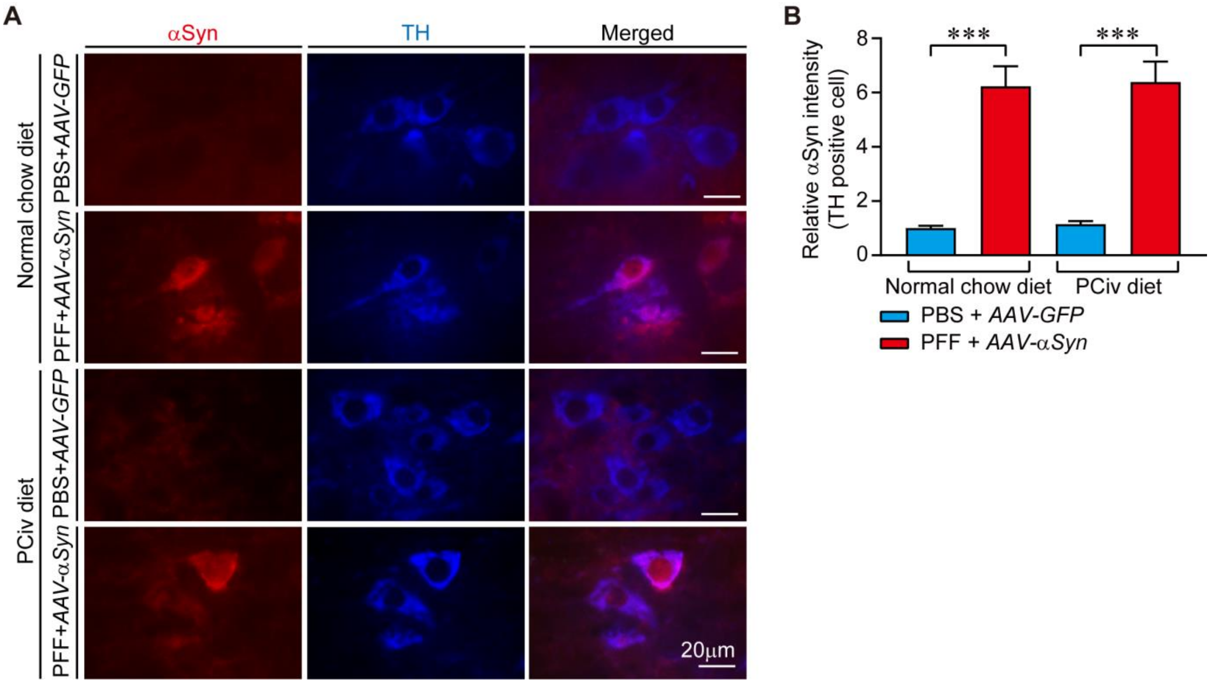

Supplementary Figure S4. Total α-synuclein expression is not altered with PCiv treatment *in vivo*

(**A**) Representative immunofluorescence images of αSyn and TH in the substantia nigra coronal sections of the indicated experimental mice groups. (**B**) Quantification of relative αSyn fluorescence intensities in the TH-positive dopamine neurons of the substantia nigra sections from the indicated experimental groups (n = 32 cells from 5 mice per group). The quantified data are expressed as means ± SEMs. Statistical significance was determined by an ANOVA test with Tukey's post hoc analysis, ***$p < 0.001$. ns = non-significant.

Supplementary Table S1. Pharmacokinetic parameters of PCiv

Pharmacokinetic properties of PCiv obtained from the analysis of Figure 3. Cmax, maximum serum concentration; Tmax, time to peak drug concentration; AUClast and AUCinfinity, area under curve from time 0 to the last measurable concentration or infinity time point; CLt, total drug clearance from body; Vss, steady state volume of distribution; MRT, mean residence time; BA, bioavailability.

	i.v. (n=5)	p.o. (n=4)
Cmax (μg/mL)	–	0.03453 ± 0.02143
Tmax (min)	–	213.8 ± 144.1
t1/2 (min)	85.50 ± 51.36	97.22 ± 10.25
AUClast (μg/mL×min)	76.89 ± 23.29	7.44 ± 5.19
AUCinfinitiy (μg/mL×min)	78.72 ± 24.55	7.83 ± 5.28
CLt (mL/min/kg)	136.5 ± 38.5	–
Vss (mL/kg)	6620.9 ± 1899.7	–
MRT (min)	53.34 ± 26.63	–
BA (%)	–	9.96

Supplementary Table S2. PCiv tissue distribution

Detailed information on the concentration of PCiv in each tissue 15 min after intravenous administration of PCiv in rats. The tissue-to-plasma concentration ratio is shown in the last column.

Organs	Concentration (ng/ML, ng/g organ)	Tissue–to–plasma concentration ratio
Plasma	1116 ± 295 ng/ml	–
Liver	337 ± 76 ng/g tissue	0.32 ± 0.13
Heart	4580 ± 274 ng/g tissue	4.32 ± 1.21
Kidney	594 ± 196 ng/g tissue	0.54 ± 0.17
Brain	6790 ± 2846 ng/g tissue	6.40 ± 3.42
